# Two Novel Palbociclib-Resorcinol and Palbociclib-Orcinol Cocrystals with Enhanced Solubility and Dissolution Rate

**DOI:** 10.3390/pharmaceutics14010023

**Published:** 2021-12-23

**Authors:** Chenxin Duan, Wenwen Liu, Yunwen Tao, Feifei Liang, Yanming Chen, Xinyi Xiao, Guisen Zhang, Yin Chen, Chao Hao

**Affiliations:** 1 Jiangsu Key Laboratory of Marine Biological Resources and Environment, Jiangsu Key Laboratory of Marine Pharmaceutical Compound Screening, School of Pharmacy, Jiangsu Ocean University, Lianyungang 222005, China; 2019220308@jou.edu.cn (C.D.); 2019220322@jou.edu.cn (W.L.); 2019220319@jou.edu.cn (F.L.); 2020220606@jou.edu.cn (Y.C.); 2021226077@jou.edu.cn (X.X.); gszhang@hust.edu.cn (G.Z.); 2 Department of Chemistry, Southern Methodist University, 3215 Daniel Avenue, Dallas, TX 75275-0314, USA; yunwent@smu.edu; 3 Department of Biomedical Engineering, College of Life Science and Technology, Huazhong University of Science and Technology, Wuhan 430074, China

**Keywords:** palbociclib, cocrystal, Hirshfeld surface, solubility, dissolution rate, physical stability

## Abstract

Palbociclib (PAL) is an effective anti-breast cancer drug, but its use has been partly restricted due to poor bioavailability (resulting from extremely low water solubility) and serious adverse reactions. In this study, two cocrystals of PAL with resorcinol (RES) or orcinol (ORC) were prepared by evaporation crystallization to enhance their solubility. The cocrystals were characterized by single crystal X-ray diffraction, Hirshfeld surface analysis, powder X-ray diffraction, differential scanning calorimetry, thermogravimetric analysis, Fourier transform infrared and scanning electron microscopy. The intrinsic dissolution rates of the PAL cocrystals were determined in three different dissolution media (pH 1.0, pH 4.5 and pH 6.8), and both cocrystals showed improved dissolution rates at pH 1.0 and pH 6.8 in comparison to the parent drug. In addition, the cocrystals increased the solubility of PAL at pH 6.8 by 2–3 times and showed good stabilities in both the accelerated stability testing and stress testing. The PAL-RES cocrystal also exhibited an improved relative bioavailability (1.24 times) than PAL in vivo pharmacokinetics in rats. Moreover, the in vitro cytotoxicity assay of PAL-RES showed an increased IC_50_ value for normal cells, suggesting a better biosafety profile than PAL. Co-crystallization may represent a promising strategy for improving the physicochemical properties of PAL with better pharmacokinetics.

## 1. Introduction

Improving the solubility of poorly water-soluble drugs is one of the current challenges facing the pharmaceutical industry [1]. The core of this endeavor underlies the exploitation of different forms of APIs, such as cocrystals, amorphous solids [2], polymorphs [3], salts [4], hydrates [5], and solvent compounds [6]. Among these, cocrystals have garnered increasing attention for their pragmatic role in manipulating solid state properties for drug development and product reformulation [7,8]. Drug cocrystals refer to crystalline substances composed of two or more different molecules, usually with active pharmaceutical ingredients (API) and cocrystal formations (“coformers”) in the same crystal lattice [9,10]. Drug cocrystals retain API‘s pharmacological properties and can improve the physical and chemical properties of the original drug, including the melting point [11,12], solubility [13,14,15], dissolution rate [16,17,18,19], bioavailability [20,21], stability [22], and mechanical properties [23,24]. Moreover, different coformers can be selected according to the different properties of drugs to prepare cocrystals, which provides a broader space for the development of pharmaceutical preparations [25,26,27,28].

**Figure 1:**
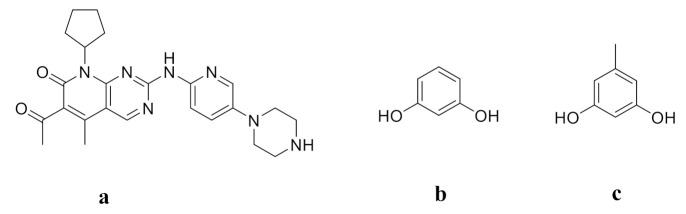
Molecular Structures of (**a**) PAL, (**b**) RES, and (**c**) ORC.

Palbociclib (PAL, Figure 1a) is an antitumor drug developed by Pfizer to treat estrogen (HR)-positive and human epidermal factor (HER2)-negative breast cancer. It is the first CDK4/6 inhibitor approved for cancer treatment [29]. PAL has also been found to be effective against non-small cell lung cancer, lymphoma, and multiple myeloma [30,31,32]. Due to its effectiveness and high selectivity for CDK4/6, PAL has become a heavyweight clinical drug for the treatment of breast cancer [33,34]. However, the pH-dependent solubility of PAL greatly limits its oral absorption and bioavailability [35]. Moreover, the long-term oral administration of PAL may lead to bone marrow toxicity, neutropenia, infection, fatigue, nausea, and stomatitis [36,37]. In order to improve the oral bioavailability of PAL and thus to decrease dosage and toxicity, some methods have been explored, such as adding excipients, changing the crystal form or preparing co-amorphous drug systems [38,39,40,41]. Zhang et al. reported that the co-amorphous PAL-acid systems greatly improved the solubility and dissolution of PAL [41]. However, there has been no study involving co-crystallization techniques to enhance the solubility of PAL. Resorcinol (RES, Figure 1b) and orcinol (ORC, Figure 1c) are two commonly used coformers to form cocrystals with APIs to improve the solubility and dissolution rate [42,43,44,45,46]. Cho et al. reported that the maximum aripiprazole concentration of the ARI-RES and ARI-ORC cocrystals were 2.6 mg/L and 6.4 mg/L respectively, which showed that these cocrystals exhibit solubility enhancement (ARI: 1.6 mg/L) [42]. Bofill et al. reported that the dissolution rate of vitamin D_3_-RES cocrystal was about two times higher than that of pure vitamin D_3_ [46].

In this study, in order to improve the solubility and dissolution rate of PAL, we choose two water-soluble coformers RES and ORC to prepare cocrystals. The obtained cocrystals were characterized by various spectroscopic techniques, and their crystal structures and intermolecular interactions were further analyzed by Hirshfeld surface and molecular electrostatic potential. The dissolution behaviors of the two cocrystals were studied under different pH media, and their biopharmaceutical profiles were evaluated through pharmacokinetics and cytotoxicity assay. To the best of our knowledge, this is the first report on improving the solubility, bioavailability, and biosafety of PAL through co-crystallization techniques, which may represent a promising strategy for improving the physicochemical properties of PAL with better pharmacokinetics.

## 2. Materials and Methods

### 2.1. Materials

Palbociclib (purity ≥ 99%) was obtained from Shanghai Aladdin Bio-Chem Technology Co., Ltd. (Shanghai, China). Resorcinol (purity ≥ 99%) was purchased from Macklin Biochemical Technology Co., Ltd. (Shanghai, China). Orcinol (purity ≥ 98%) was supplied from Energy Chemical Co., Ltd. (Shanghai, China). All the substances were employed without further purification.

### 2.2. Cocrystals Preparation

The cocrystals of PAL-RES and PAL-ORC were synthesized by the slow evaporation method. PAL (447.5 mg, 1.0 mmol) was dissolved with 100 mL dichloromethane, and the cocrystal former was dissolved in other solvents (1.0 mmol RES in 4 mL acetonitrile; 1.0 mmol ORC in 4 mL methyl isobutyl ketone). The solution of PAL was filtered through a 0.45 μm PTFE filter into an eggplant-shaped bottle, and then the coformer solution was also filtered into the same bottle without stirring or shaking. The mixture was placed in the open air at room temperature (20 ± 5 °C, 15 ± 5% RH) for 3–4 days, and the precipitated cocrystals were washed with heptane for X-ray diffraction analysis.

### 2.3. Single Crystal X-ray Diffraction (SCXRD)

Under 100 K, the single crystal X-ray diffraction (SCXRD) data of PAL-RES was collected by XtaLAB PRO diffractometer (graphite monochromatic CuKα radiation, λ = 0.154178 Å) (Riga Library, Tokyo, Japan). The PAL-ORC Single Crystal X-ray Diffraction (SCXRD) data were recorded on a Bruker Apex2 CCD diffractometer at 104.15 K (graphite monochromated MoKα radiation, λ = 0.71073 Å) (Karlsruhe, Germany). The integration and scaling of the intensity data were performed using the CrystalClear program. The crystal structures were analyzed by using the direct method in Shelxs-13 and refined using Shelxl-97 [47]. All non-hydrogen atoms were refined by using the full matrix least squares method.

### 2.4. Hirshfeld Surface Analysis

The structures of PAL cocrystals were also investigated via the Hirshfeld surfaces analysis. The 2D fingerprint plots, close contacts contributions, surfaces mapped over d_norm_, and shape index were performed by using Crystal Explorer 17.

### 2.5. Molecular Electrostatic Potential Analysis

The molecular electrostatic potential (MEP) of the parent compounds and the cocrystals was computed by the density functional theory (DFT) methods employing the def2-SVP basis set and B3LYP functional with ORCA 5.0 package [48].

### 2.6. Powder X-ray Diffraction (PXRD)

The X’PERT POWDER X-ray diffractometer (PANalytical, Holland) was used to collect the powder X-ray diffraction (PXRD) of samples in the θ/2θ scanning mode with Cu-Kα radiation (λ = 1.540598Å). The measurements were performed at room temperature, at a scan rate of 2°/min over a 2θ range of 4° to 50°. Silicon was used as an external calibrator. The PXRD of cocrystals were simulated by Mercury 4.3.0 at λ = 1.54056 Å, at a scan rate of 2°/min over a 2θ range of 5° to 50°. 

### 2.7. Thermal Analyses

Differential scanning calorimetry (DSC) analysis was carried out with Netzsch DSC 200F3 equipment (Netzsch, Selb, Germany), and the samples were heated (10 °C /min from 30 to 300 ℃) in a N_2_ atmosphere (flow rate 40 mL/min). For the DSC analysis, a typical sample weighing 3–5 mg was used.

On the Netzsch TG 209F3 equipment (Netzsch, Selb, Germany), thermogravimetric analysis (TGA) was performed at a scan rate of 10 °C/min at 30 to 300 °C under a flow of nitrogen (20 mL/min). Approximately 4–6 mg of sample was used each time.

### 2.8. Attenuated Total Reflection Fourier’s Transform Infrared Spectroscopy (ATR-FTIR)

A Fourier transform infrared spectrometer (Vertex 70, Brooke, Billerica, MA, USA) was used for the infrared spectroscopy. In addition, an attenuated total reflectance (ATR) accessory was used, and a Zn-Se crystal was used to verify the spectrum compared to the sample. The scanning range was from 500 cm^−1^ to 4000 cm^−1^ with a resolution of 2 cm^−1^.

### 2.9. Scanning Electron Microscopy (SEM)

The morphologies of the crystals were recorded by scanning electron microscopy (SEM) on a Phenom Pro desktop scanning electron microscope (Eindhoven, The Netherlands). A sputter coating apparatus was applied to induce electric conductivity on the surface of the samples using a gold sputter module. The source of the electron beam was CeB6 electron source, and the vacuum degree was 1.31e−003 Pa. The images were taken by the detector of back scattered electrons (BSE); the acceleration and probe voltage were set to 5 kV. The magnifications of the analyses of the samples were PAL-RES in 250×, PAL-ORC in 500×, PAL in 2000×, RES in 500× and ORC in 200×.

### 2.10. Intrinsic Dissolution Rate (IDR) Experiments

About 100 mg of the sample was weighed, pressed, and punched into a diameter of 9.0 mm into a compact that did not disintegrate. In the IDR experiment, one side of the compact was sealed with melted wax, and the other side was not covered to contact with the solution. The temperature was set to 37 ℃, the paddle speed was 100 rpm, the samples were collected at the corresponding time points (phosphate buffer pH 6.8: 60, 120, 180, 240, 300, 360 min; acetic acid buffer pH 4.5: 1, 2, 3, 5, 7, 10 min; hydrochloric acid solution pH 1.0: 10, 20, 30, 40, 50, 60 s) and supplemented with the same volume of culture base. Filtered the supernatant (0.22 μm) to remove undissolved particles. The PAL concentration was measured by an LC-20AD HPLC system (Shimadzu, Kyoto, Japan). At 40 °C, an Agilent ZORBAX Eclipse XDB-C_18_ column (250 mm × 4.6 mm, 5 μm, Agilent, Santa Clara, CA, USA) and a UV detector with a wavelength of 356 nm were used to separate the palbociclib. The mobile phase contained 0.1% acetic acid in methanol and water (80:20, *v*/*v*), the flow rate was 0.8 mL/min, and the isocratic elution was 10 min. The results were stated as the average of three replicated experiments.

### 2.11. In Vitro Dissolution Studies

According to the Chinese Pharmacopoeia of the paddle method, the powder was dissolved on a FADT-1202RC automatic sampling dissolution instrument (Shanghai Fox Analytical Instrument Co., Ltd., Shanghai, China) in 500 mL pH 6.8 phosphate buffer at 37 ± 0.5 °C In the experiment, the stirring speed was 50 rpm. PAL, PAL-RES, and PAL-ORC were ground and passed through a 200 mesh sieve. In total, 50 mg of PAL or a PAL equivalent amount of cocrystal were weighed for the powder dissolution experiment. At 5, 10, 15, 30, 45, 60, 90, 120, 180, 240, 300, 360, 480, 600, 720, 1080, 1440 min, taken out 1 mL of the dissolved sample and incubated with an equal volume of fresh medium in order to maintain a constant dissolution system. The PAL concentration was measured using the same method as the IDR part. All the experiments were conducted in triplicate.

### 2.12. Stability Studies

For the physical stability studies, the powder samples of the cocrystals were exposed to accelerated condition (YSEI, China) with 40 °C/75% RH for three months; subsequently, the samples were analyzed by PXRD.

The cocrystals were subjected to a high temperature (60 °C), high humidity (92.5% RH), and strong illumination (4500 lx) condition respectively for stress testing experiments. The samples were analyzed by PXRD to detect possible solid-state transformation at 0, 5, 10 days.

### 2.13. In Vivo Pharmacokinetic (PK) Experiments

The PK profiles of PAL, PAL-RES and PAL-ORC were determined in adult male Sprague−Dawley rats (SD rats, 200–250 g), which were commercially obtained from Pizhou Oriental Breeding Co., Ltd. (Xuzhou, China). All the experiments were approved by the Ethics and Experimental Animal Committee of Jiangsu Ocean University (Project identification code: 202100021). The rats were housed in standard cages with constant temperature and humidity, and a 12 h light−dark cycling condition was set. Before the commencement of the studies, all the rats were fasted overnight with free access to water for 12 h. Fifteen animals were randomly assigned to three groups (*n* = 5/group) and intragastrically administrated with PAL, PAL-RES, and PAL-ORC in 0.5% (*w*/*v*) aqueous sodium methyl cellulose solution, respectively. A dose equivalent to 5 mg/kg body weight of PAL was used to conduct the evaluation. After the oral administration, blood collections were performed at designated time points (0.5, 1, 2, 4, 6, 8, 12, 24 and 48 h) using centrifuge tubes containing EDTA-K2. Next, the plasma samples were obtained from the whole blood by centrifuging at 4000 rpm for 10 min. The plasma samples were prepared for HPLC analysis by washing with 300 μL methanol, and the PAL concentration was measured using the same method as described in “2.10. Intrinsic dissolution rate (IDR) experiments”. Moreover, the PK parameters were calculated by Phoenix WinNonlin 6.0 pharmacokinetic software package (Certara, Princeton, NJ, USA).

### 2.14. In Vitro Cytotoxicity Assay

The human umbilical vein endothelial cell line (HUVEC) was a gift from Prof. F Y Chen (Xiangya Hospital, Changsha, China). The cells were grown in DMEM (Hyclone, Logan, UT, USA) supplied with 10% fetal bovine serum (Hyclone, Logan, UT, USA) and 1% penicillin/streptomycin mixture. Furthermore, all the cells were cultured at a 37 ℃, 5% CO_2_ humidified incubator.

The cytotoxicity activity of compounds PAL, PAL-RES and PAL-ORC for normal HUVEC cell line was assessed via the MTT assay. The cells were cultured at a density of 8000 cells/well in a 96 well plate. Five different concentrations (0, 0.1, 1, 10 and 100 μM) of compounds PAL, PAL-RES and PAL-ORC in DMSO were subsequently added to the wells then incubated under 5% CO_2_ at 37 °C for 48 h; 50 μL of MTT (2 mg/mL) was added to each well, and the cells were incubated for another 4 h. Next, the liquid in each well was removed, and DMSO (150 μL) was added. The absorbance (OD values) at 490 nm was measured by a microplate reader (Biotek, SYNERGY HTX, VT, USA). Each concentration was tested in triplicate.

## 3. Results

### 3.1. Single-Crystal X-ray Diffraction (SCXRD) 

By using the mixed solution of dichloromethane with acetonitrile or methyl isobutyl ketone as the crystallization solvent, we successfully obtained the yellow clustered crystals of PAL-RES and PAL-ORC. The cocrystal data were collected by SCXRD to obtain the three-dimensional (3D) structure and the intermolecular interactions of the cocrystals. The corresponding crystallographic data and refinement details are given in Table 1, and the hydrogen bond geometry is provided in Table 2.

**Figure 2:**
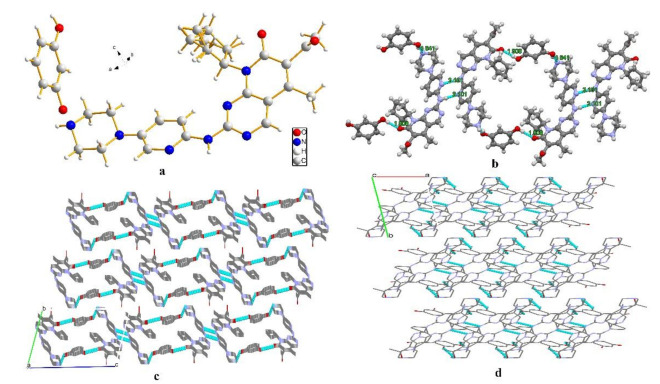
(**a**) Asymmetric unit of PAL-RES, (**b**) 1D hydrogen bonds chain structure of PAL-RES, (**c**) 3D hydrogen-bonded frameworks of PAL-RES along a-axis, (**d**) 3D hydrogen-bonded frameworks of PAL-RES along c-axis.

**Figure 3:**
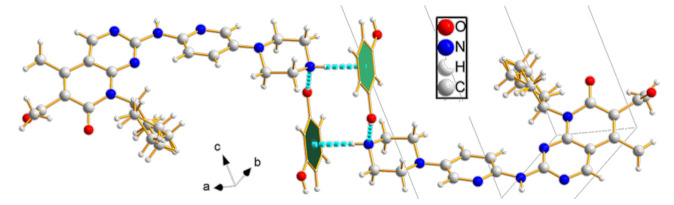
N-H· · ·π (2.30 Å) interaction between PAL-RES molecules.

PAL-RES crystallized in the triclinic space group P-1, with one molecule each of PAL and RES in the asymmetric unit (Figure 2a). The lattice parameters were as follows: a = 6.63890 Å, b = 12.4152 Å, c = 18.2788 Å, α = 73.114°, β = 81.7870°, and γ = 78.1760°. The PAL and RES molecules were connected through an O3-H3·N7 (1.841 Å, 159.12°) hydrogen bond to form a dimer. Two further dimers were connected by an O4-H4C· · ·O1 (1.908 Å, 177.59°) hydrogen bond between the hydroxyl group in RES and the carbonyl group in PAL. The dimers were held together through an N4-H4·N5 (2.101 Å, 178.20°) hydrogen bond between amino groups of neighboring PAL molecules to generate a one-dimensional (1D) belt (Figure 2b). The belts were further stacked along the a-axis to form a 3D hydrogen-bonded framework structure (Figure 2c). As shown in Figure 2d, the stacking along the c-axis formed a 3D hydrogen bond frame structure. Moreover, N-H· · ·π interactions were observed between PAL-RES molecules (Figure 3). 

**Figure 4:**
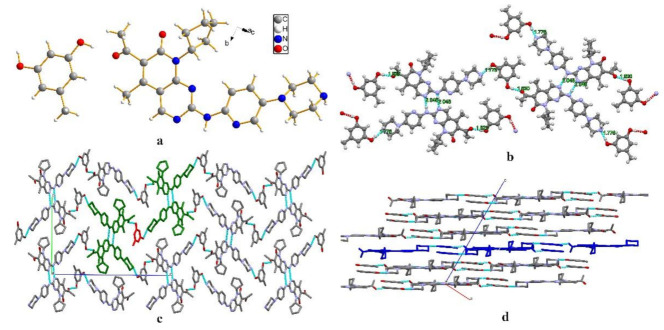
(**a**) Asymmetric unit of PAL-ORC, (**b**) 1D hydrogen bonds chain structure of the PAL-ORC cocrystal, (**c**) 3D hydrogen-bonded frameworks of PAL-ORC along a-axis, (**d**) 3D hydrogen-bonded frameworks of PAL-ORC along b-axis.

PAL-ORC crystallized in the monoclinic space group P21/c, with one molecule each of PAL and ORC in the asymmetric unit (Figure 4a). The lattice parameters were as follows: a = 6.5309 Å, b = 16.8792 Å, c = 25.8097 Å, α = 90°, β = 92.819°, and γ =90°. PAL and ORC molecules were connected through an O3-H3·O1 (1.820 Å, 178.04°) hydrogen bond to form a dimer. Two dimers were further formed by N4-H4·N5 (2.048 Å, 173.05°) hydrogen bond between amino groups of neighboring PAL molecules. The dimers were held together through an O4-H4AB·N2 (1.776 Å, 174.11°) hydrogen bond to generate a one-dimensional (1D) belt (Figure 4b). The belts were further stacked along a-axis to form a 3D hydrogen-bonded framework structure (Figure 4c). Figure 4d shows the stacking along the b-axis to form a 3D hydrogen bond frame structure.

### 3.2. Hirshfeld Surfaces and 2D Fingerprint Plots 

**Figure 5:**
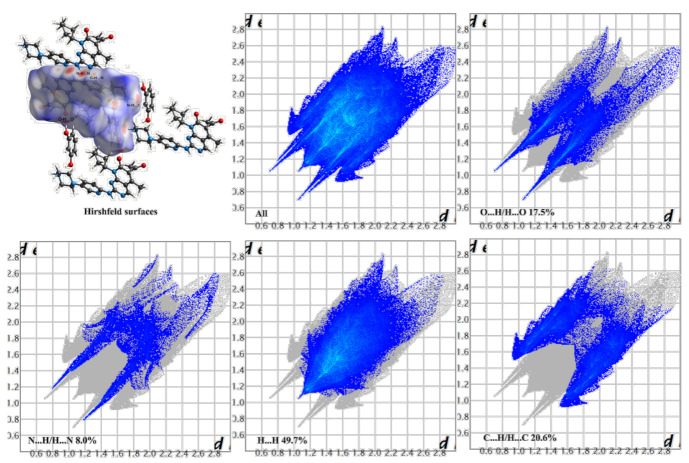
Hirshfeld surface and 2D fingerprint plots of PAL-RES.

**Figure 6:**
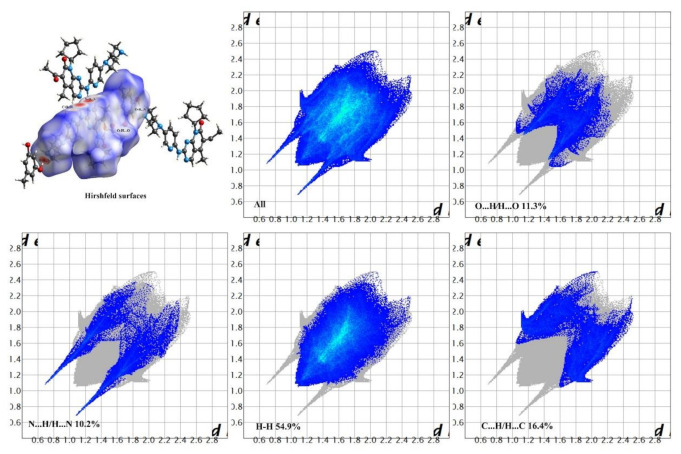
Hirshfeld surface and 2D fingerprint plots of PAL-ORC.

**Figure 7:**
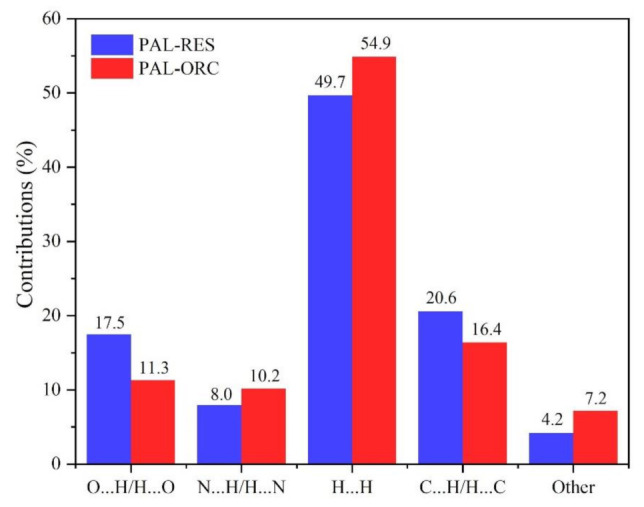
Relative contributions to the Hirshfeld surface areas of PAL-RES and PAL-ORC.

Hirshfeld surface analysis is a tool to investigate the packing modes and the interactions between molecules in crystals [49]. To gain further insight into the intermolecular interaction of the PAL-RES/PAL-ORC cocrystal, Hirshfeld surfaces (HS) and the fingerprint plots were plotted by using Crystal Explorer 17. As illustrated in Figure 5 and Figure 6, the red areas on the surface correspond to the shortest N–H·N, C–H·N, O–H·O, and O–H···N interactions, while the blue areas on the surfaces represent H···H contacts. Overall, the HS of PAL-RES and PAL-ORC differ from each other in shape, reflecting the different proportions of intermolecular contacts. For a clearer quantitative comparison, the percentage contributions of the surface or fingerprints of the main interaction diagrams of PAL-RES and PAL-ORC were summarized as the histogram shown in Figure 7. The quantitative analysis (Figure 7) showed that the H···H interactions were the largest contributors to the total HS for PAL-RES and PAL-ORC compared to other contacts, indicating that the cocrystal structurally packs loosely, which creates favorable conditions to regulate the dissolution capability of PAL [50].

In addition, the H·O/O·H contacts corresponded to 17.5% of all HS in the PAL-RES and can be easily identified as two symmetrical spikes. But in PAL-ORC, the H···N/N···H contacts were the most powerful intermolecular interaction force, accounting for 10.2% of the d_norm_ surface. The different contributions of N-H···O/O-H···O and H···H/C···H in two cocrystals may have been caused by the varied positions of hydrogen bonds and coformers (Figure 7, Figures S1 and S2). Finally, there were N-H···π (Figure 3, 2.33 Å) and C-H···π (Figure S3) between PAL-RES molecules, and there were not only C-H···π (Figure S4) but also π···π (Figure S5) between PAL-ORC molecules.

### 3.3. Molecular Electrostatic Potential Analysis 

**Figure 8:**
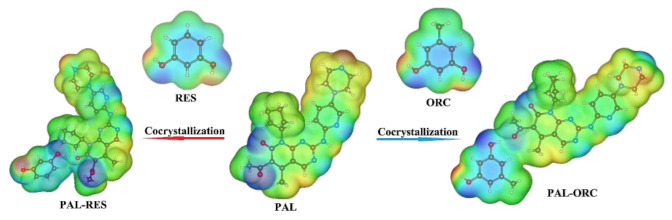
Molecular electrostatic potential maps of PAL, RES, ORC, PAL-RES, and PAL-ORC.

Molecular electrostatic potential (MEP) is a powerful tool to investigate the noncovalent interactions of molecules and the distribution of electronic charge within a cocrystal [51]. To gain more information about the nucleophilic or electrophilic regions of PAL-RES/PAL-ORC, the molecular electrostatic potential surface map was created (Figure 8). The blue- and red-colored regions indicate the negative electrostatic potential (nucleophiles) and positive electrostatic potential (electrophile), respectively. The green region shows neutral potential. As expected, the negative charges were located on the oxygen atoms, and the most positive regions of the molecules are the regions where the hydrogen bonded to oxygen and nitrogen atoms are located due to the partial positive charge δ+ on the hydrogens. The carbonyl group in the PAL molecule formed hydrogen bond interactions with the hydroxyl group of the RES or ORC, which was consistent with the SXRD analysis and Hirshfeld surface analysis.

### 3.4. Powder X-ray Diffraction Analysis (PXRD) 

**Figure 9:**
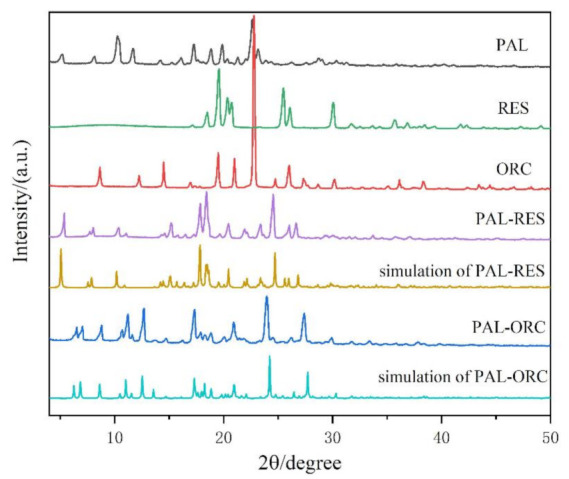
Powder X-ray diffraction (PXRD) patterns of PAL, RES, ORC, PAL-RES (experimental and simulated) and PAL-ORC (experimental and simulated).

The powder X-ray diffraction characterization of PAL, RES, ORC, PAL-RES, and PAL-ORC was performed. The results are shown in Figure 9. PAL shows the major characteristic peaks at 2θ of 10.32°, 11.71°, and 22.68°. The characteristic peaks of RES are at 19.59°, 20.34°, 25.44°, 26.07°, and 30.06°, while there are some new featured peaks (5.42°, 15.19°, 17.84°, 18.38°, 24.53°, and 26.65°) observed in the pattern of PAL-RES. The characteristic peaks of ORC are at 8.64°, 14.49°, 19.46°, 20.95°, 22.78°, and 26.01°, while the characteristic peaks of PAL-ORC are at 6.28°, 6.80°, 11.00°, 12.47°, 17.10°, 23.82°, and 27.23°. These characteristic peaks are basically consistent with those simulated from single crystal data, confirming the crystalline phase purity of the synthesized cocrystals. 

### 3.5. Thermal Analysis

**Figure 10:**
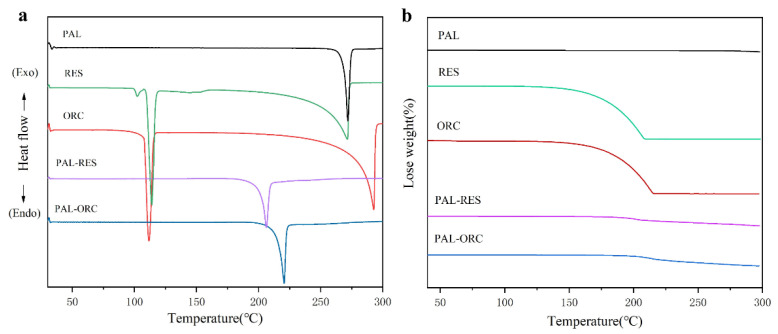
(**a**) DSC thermograms of PAL, RES, ORC, PAL-RES, and PAL-ORC, (**b**) thermogravimetric analysis (TGA) curves of PAL, RES, ORC, PAL-RES, and PAL-ORC.

Thermal analysis technology is usually employed to confirm the formation of pure solid phases due to its higher sensitivity [52]. To investigate the thermal behavior of PAL-RES and PAL-ORC in relation to the individual components, DSC and TGA analyses were performed; the results are presented in Figure 10. The endothermic peak of PAL was at 271.8 °C, which corresponds to its melting process. Both RES (113.9 °C and 271.2 °C) and ORC (111.7 °C and 292.6 °C) showed two endothermic events. The former was the melting endothermic peak while the latter corresponded to the decomposition process. The endothermic peaks of PAL-RES and PAL-ORC were at 206.2 °C and 220.3 °C, respectively. The DSC thermograms of two cocrystals were distinctly different from that of the individual components, which indicates the existence of a new crystalline solid form. From the correlation between melting point and structure, this may have been due to molecular rigidity and intermolecular interactions. Meanwhile, the TG thermograms of PAL-RES and PAL-ORC showed no weight loss below their melt points and indicated that these two cocrystals did not contain any solvents in the crystal lattice, which was also evidenced by the SCXRD analysis.

### 3.6. Attenuated Total Reflection Fourier’s Transform Infrared Spectroscopy (ATR-FTIR) 

**Figure 11:**
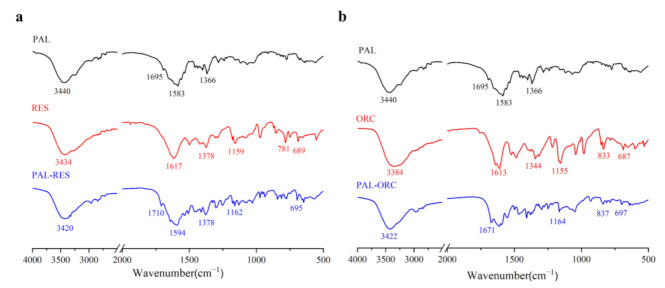
IR spectra of (**a**) PAL, RES, and PAL-RES, (**b**) PAL, ORC, and PAL-ORC.

ATR-FTIR is a fast and non-destructive characterization method which can further identify the intermolecular interactions of functional groups involved by the changes in their vibrational frequencies [44,45,46]. PAL showed characteristic peaks at 1695 and 3440 cm^−1^ (Figure 11), which were assigned to the carbonyl C=O and N−H stretching vibrations, respectively. The stretching vibration peak of -OH in RES occurred at 3434 cm^−1^, and ORC occurred at 3384 cm^−1^. After the formation of PAL-RES, the C=O stretching vibrations were observed at 1710 cm^−1^, and the O−H and N−H stretching shifted to 3420 cm^−1^. The carbonyl group of PAL-ORC was observed at 1671 cm^−1^ and the O−H and N−H stretching were observed at 3422 cm^−1^. These variations of the characteristic peaks can be attributed to the new formation of intermolecular interactions of PAL and RES/ORC. It should be mentioned that these interactions can be described as hydrogen bonds, because the shifts did not exceed 50 cm^−1^ [53].

### 3.7. Scanning Electron Microscopy (SEM) Analysis 

**Figure 12:**
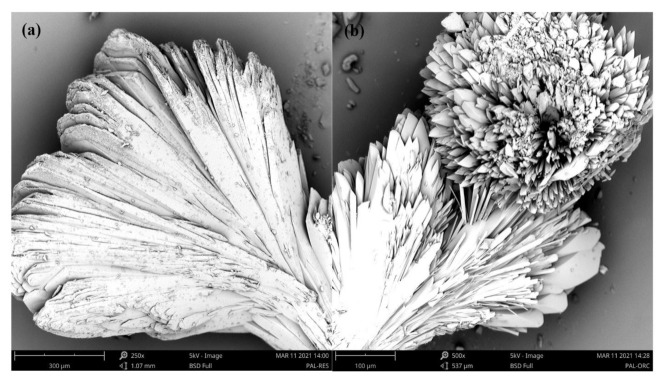
SEM images of (**a**) PAL-RES in 250×, and (**b**) PAL-ORC in 500×.

The crystal habit of API is an important parameter in the pharmaceutical process, which affects various pharmaceutical parameters, such as the flow characteristics and compaction characteristics of the drug powder [54]. In Figure 12 and Figures S6–S8, the SEM images of PAL, RES, ORC, PAL-RES, and PAL-ORC are shown. The crystals of PAL were needle-shaped and easily cluster into clusters. While the surface of the RES and ORC crystals was smooth, the RES crystals were columnar particles, and the ORC crystals were bulk material. Both PAL-RES and PAL-ORC were cluster particles.

### 3.8. Intrinsic DissolutionRate (IDR) Experiments

**Figure 13:**
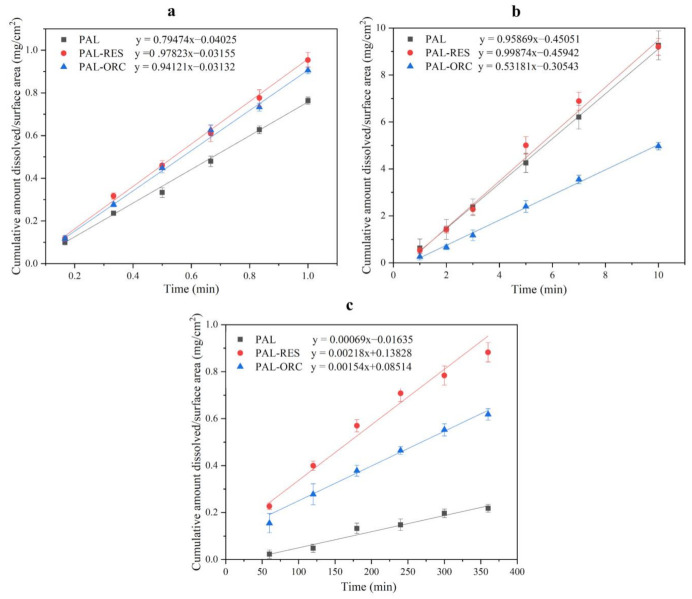
IDR curves of PAL, PAL-RES, and PAL-ORC, (**a**) in hydrochloric acid solution (pH 1.0), (**b**) in acetate buffer pH 4.5, (**c**) in phosphate buffer pH 6.8.

IDR values are generally used to predict the bioavailability of the investigated samples [28]. The results of the IDR experiment are shown in Figure 13. In the hydrochloric acid solution (pH 1.0), the order of the dissolution rate was PAL-RES > PAL-ORC > PAL (Figure 13a). Compared with PAL, the dissolution rate of PAL-RES and PAL-ORC was 1.2 times faster. In the pH 4.5 acetate buffer, the dissolution rate was PAL-RES > PAL > PAL-ORC (Figure 13b). The PAL-RES showed only a slight increase in dissolution rate, while PAL-ORC showed the lowest release rate, at 0.53 mg/min/cm^2^. In the pH 6.8 phosphate buffer, the order of dissolution rates was: PAL-RES > PAL-ORC > PAL (Figure 13c). The dissolution rate of PAL-RES and PAL-ORC increased by three times and two times, respectively.

### 3.9. Powder Dissolution Analysis 

**Figure 14:**
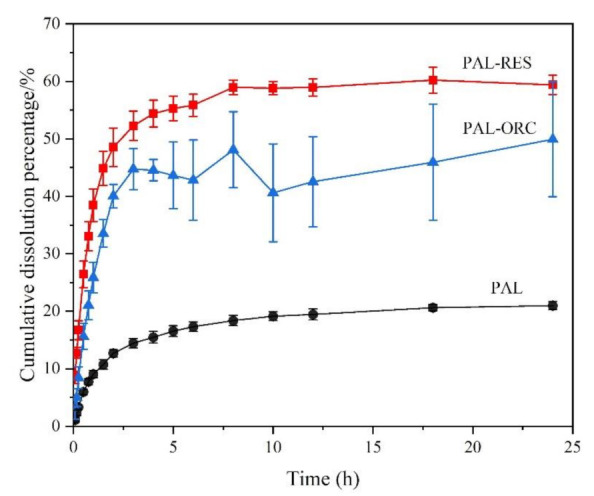
Powder dissolution profile of PAL, PAL-RES, and PAL-ORC in phosphate buffer pH 6.8.

PAL has good solubility under acidic conditions and dissolves completely at pH 1.0 or 4.5 within five minutes. Therefore, the powder dissolution analysis of two cocrystals was only evaluated at pH 6.8. The dissolution profiles of PAL-RES and PAL-ORC are shown in Figure 14. The rank order of the cumulative amount dissolved of PAL was PAL-RES > PAL-ORC > PAL. Compared with API, the dissolution of PAL-RES and PAL-ORC was significantly improved. The equilibrium dissolution of PAL-RES and PAL-ORC was 60.22% and 49.93%, respectively, while the equilibrium dissolution of PAL was only 20.09%. The results agreed well with the results of the IDR experiment. The dissolution analysis and IDR results indicated that both PAL-RES and PAL-ORC presented a better physiochemical property than PAL, which may result in an improved bioavailability.

### 3.10. Stability 

**Figure 15:**
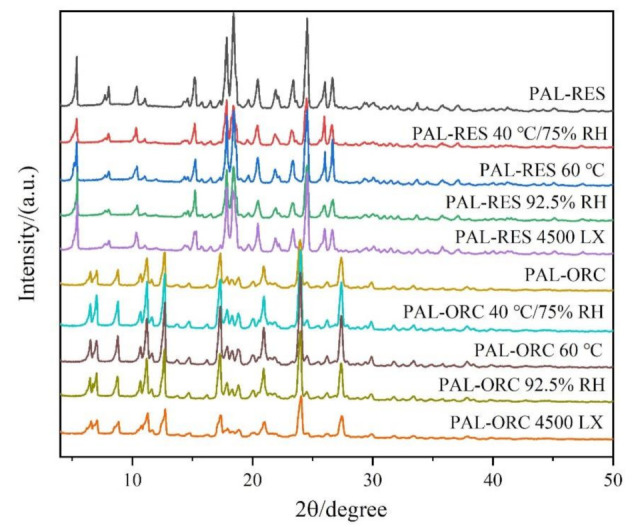
PXRD patterns of PAL-RES and PAL-ORC under stress testing conditions (10 days) and accelerated conditions (3 months).

The physical stabilities of PAL-RES and PAL-ORC were investigated under accelerated stability testing and the stress testing. As illustrated in Figure 15, the PXRD analysis revealed that the PXRD patterns of PAL-RES and PAL-ORC all remained the same compared with the corresponding initial cocrystals without phase transformation. This analysis indicated that PAL-RES and PAL-ORC were physically stable under accelerated stability conditions (40 °C/75% RH for 3 months). Moreover, the cocrystals of PAL-RES and PAL-ORC remained unchanged at high temperature (60 °C), high humidity (92.5% RH), and strong illumination (4500 LX), which indicated that they were all physically stable for at least 10 days under stress testing conditions. 

### 3.11. In Vivo Pharmacokinetic (PK) Experiments

**Figure 16:**
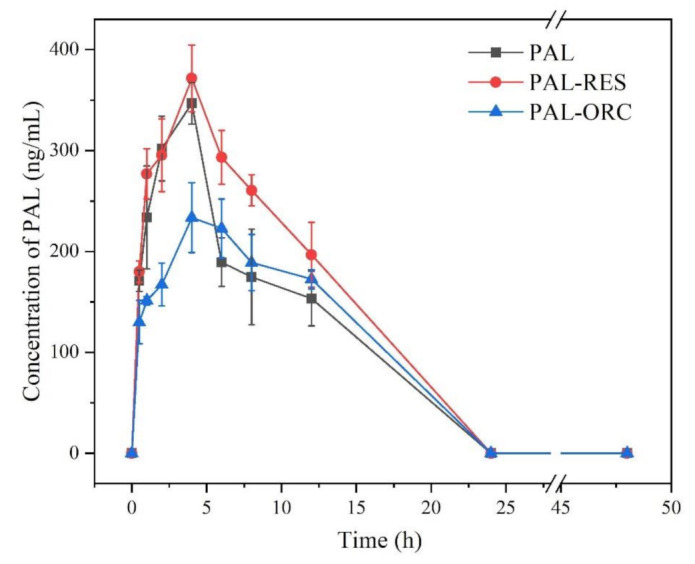
PK profiles of PAL, PAL-RES and PAL-ORC after single oral dose equivalent to 5 mg/kg body weight of PAL in SD rats.

The pharmacokinetic profiles of PAL and two cocrystals are shown in Table 3 and Figure 16. The AUC of PAL-RES (3245.47 ng∙h/mL) was higher than PAL (2616.48 ng∙h/mL), but PAL-ORC decreased to 2254.08 ng∙h/mL, indicating PAL-RES presented better absorption in rats than PAL. The relative bioavailability of the cocrystals PAL-RES and PAL-ORC was found to be 1.24 and 0.86 times, respectively, with respect to the parent drug (Table 3). As shown in Figure 16, the C_max_ of PAL in PAL-RES (371.57 ng/mL) was higher than that of PAL (346.69 ng/mL), and the C_max_ of PAL in PAL-ORC (233.46 ng/mL) was lower than that of PAL. Lower V_d_ value means more of the drug is contained in plasma; by contrast, higher V_d_ values means the drug binding more to certain tissues more [55]. The V_d_ values of PAL-RES and PAL-ORC were 12.05 and 19.26 L/kg respectively. The results indicated that PAL-RES showed a better plasma distribution in comparison with PAL and PAL-ORC. 

The above results of the pharmacokinetic studies coincide with the results of the in vitro IDR and powder dissolution experiments, showing that the oral bioavailability of PAL can be indeed improved via the formation of a PAL-RES cocrystal.

### 3.12. In Vitro Cytotoxicity Study 

Considering the potential clinical use of the obtained cocrystal, an in vitro cytotoxicity study against a normal cell line of HUVEC was performed. The cytotoxicity of both PAL-RES and PAL-ORC was concentration-dependent. RES and ORC exhibited negligible cytotoxicity (IC_50_ > 100 μM) toward HUVEC after culturing the cells with the drugs for 48 h. The calculated IC_50_ value of PAL-RES was 4.02 ± 0.07 μM, which was higher than that of PAL (IC_50_ = 2.40 ± 0.07 μM), suggesting that PAL may feature a better biosafety profile after forming a cocrystal with RES. Unfortunately, PAL-ORC (IC_50_ = 0.85 ± 0.13 μM) had more potent cytotoxicity against HUVEC.

## 4. Conclusions

In this study, two novel cocrystals of PAL with RES and ORC were prepared using the solvent evaporation method. The cocrystals were characterized by single-crystal and powder X-ray diffraction, differential scanning calorimetry, thermogravimetric analysis, Fourier transform infrared and scanning electron microscopy. The intermolecular interactions of cocrystals were further analyzed by HS and fingerprint spectra. In the pH 6.8 phosphate buffer, the in vitro dissolution rate of PAL-RES and PAL-ORC increased by three times and two times, respectively, which may have been due to their rich hydrogen bond interactions and sandwich structure leading to rapid dissolution and diffusion. Meanwhile, the cocrystals showed great physical stability in accelerated stability testing and stress testing. In the in vivo PK and in vitro cytotoxicity experiments, PAL-RES exhibited better bioavailability and biosafety than PAL. Therefore, PAL-RES may be a potential candidate with better solubility and bioavailability, and is worthy of further study. The use of the bio-compatible and non-toxic product as a co-delivery reagent may point to new directions in pharmaceutical co-crystallization.

## Figures and Tables

**Table 1:** Crystallographic data for the refinement of the cocrystals.

Compounds	PAL-RES	PAL-ORC
Empirical formula	C_30_H_35_N_7_O_4_	C_31_H_37_N_7_O_4_
Formula weight	557.65	571.67
Temperature/K	100.00 (10)	104.15
Crystal system	triclinic	monoclinic
Space group	P-1	P21/c
a/Å	6.63890 (10)	6.5309 (2)
b/Å	12.4152 (2)	16.8792 (4)
c/Å	18.2788 (3)	25.8097 (6)
α/°	73.114 (2)	90
β/°	81.7870 (10)	92.819 (2)
γ/°	78.1760 (10)	90
Volume/Å^3^	1405.46 (4)	2841.72 (13)
Z	2	4
ρ_calc_g/cm^3^	1.318	1.336
μ/mm^−1^	0.732	0.091
F(000)	592.0	1216.0
Radiation	CuKα (λ = 0.154178)	MoKα (λ = 0.71073)
2θ/°	5.072 to 151.986	2.884 to 58.07
Goodness-of-fit on F^2^	1.064	1.067
Final R indexes [I ≥ 2σ (I)]	R_1_ = 0.0456, wR_2_ = 0.1190	R_1_ = 0.0417, wR_2_ = 0.1005
Final R indexes [all data]	R_1_ = 0.0537, wR_2_ = 0.1241	R_1_ = 0.0553, wR_2_ = 0.1064
Largest diff. peak/hole/e Å^−3^	0.30/−0.28	0.26/−0.24
CCDC NO.	2096399	2096409

**Table 2:** Hydrogen bonding table for the cocrystals.

Compounds	D-H·A	d(D-H)/Å	d(H·A)/Å	d(D·A)/Å	∠D-H·A/°	Symmetry Code
PAL-RES	O3-H3 N7	0.84	1.85	2.6455 (18)	157.5	
	O4-H4C·O1	0.84	1.91	2.7477 (15)	177.8	1 − X, 1 − Y, 1 − Z
PAL-ORC	C7-H7·N6	0.95	2.32	2.9242 (16)	120.6	
	C11-H11·N31	0.95	2.50	3.2444 (16)	135.4	2 − X, 1 − Y, 1 − Z
	C20-H20A·O2	0.99	2.60	3.1118 (18)	112.1	
	C23-H23A·O2	0.99	2.33	2.9490 (17)	119.3	
	C25-H25·O1	0.95	2.65	3.3288 (17)	128.7	
	N2-H2C·O3	0.885 (17)	2.642 (18)	3.4934 (16)	161.9 (15)	2 + X, ½ − Y, ½ + Z
	N4-H4·N5	0.88	2.05	2.9238 (14)	173.0	2 − X, 1 − Y, 1 − Z
	O3-H3·O1	0.91	1.82	2.7346 (14)	178.2	
	O4-H4AB·N23	0.99	1.78	2.7642 (15)	174.1	−3 + X, ½ − Y, −½ + Z

**Table 3:** Pharmacokinetic parameters of PAL, PAL-RES, and PAL-ORC.

	PAL	PAL in PAL-RES	PAL in PAL-ORC
C_max_ (ng/mL)	346.69	371.57	233.46
T_max_ (h)	4	4	4
T_1/2_ (h)	19.89	10.33	17.36
V_d_ (L/kg)	20.48	12.05	19.26
MRT (h)	5.36	5.73	6.15
AUC_0−t_ (ng∙h/mL)	2616.48	3245.47	2254.08
F_rel_	-	1.24	0.86

## Data Availability

The data presented in this study are contained within this article and supplementary materials.

## Supplementary Materials

Supplementary materials can be found at https://www.mdpi.com/article/10.3390/pharmaceutics14010023/s1. Figure S1: (a) C-H· · ·O interaction between PAL-RES molecules made by Mercury 4.3.0 software, (b) shape-index surface for C-H· · ·O interaction of PAL-RES through Hirshfeld Surface Analysis, Figure S2: (a) C-H· · ·O and O-H· · ·O interactions between PAL-ORC molecules made by Mercury 4.3.0 software, (b) shape-index surface for C-H· · ·O and O-H· · ·O interactions of PAL-ORC through Hirshfeld Surface Analysis. (Yellow circles: C-H· · ·O; Red circles: O-H· · ·O), Figure S3: C-H· · ·π interaction between PAL-RES molecules, Figure S4: π· · ·π interaction between PAL-ORC molecules, Figure S5: C-H· · ·π interaction between PAL-ORC molecules, Figure S6: SEM images of PAL in 2000×, Figure S7: SEM images of RES in 500×, Figure S8: SEM images of ORC in 200×.

## References

[1] Jirat J., Ondo D., Babor M., Ridvan L., Soos M. (2019). Complex methodology for rational design of Apremilast-benzoic acid co-crystallization process. Int. J. Pharm..

[2] Xm A., Sh B., Mbla C., Xu L.A., Xl C., Ysa C. (2019). Influence of mechanical and thermal energy on nifedipine amorphous solid dispersions prepared by hot melt extrusion: Preparation and physical stability—Sciencedirect. Int. J. Pharm..

[3] Guthrie S.M., Smilgies D.M., Giri G. (2018). Controlling polymorphism in pharmaceutical compounds using solution shearing. Cryst. Growth Des..

[4] Fu Q., Lu H.D., Xie Y.F., Liu J.Y., Han Y., Gong N.B. (2019). Salt formation of two bcs ii drugs (indomethacin and naproxen) with (1r, 2r)-1,2-diphenylethylenediamine: Crystal structures, solubility and thermodynamics analysis. J. Mol. Struct..

[5] RaoKhandavilli U.B., Gangavaram S., Rajesh G. (2014). High solubility crystalline hydrates of na and k furosemide salts. CrystEngComm.

[6] Boothroyd S., Kerridge A., Broo A., Buttar D., Anwar J. (2018). Why do some molecules form hydrates or solvates?. Cryst. Growth Des..

[7] Patel D.J., Puranik P.K. (2020). Pharmaceutical Co-crystal: An Emerging Technique to enhance Physicochemical properties of drugs. Int. J. Chemtech Res..

[8] Bhatt J.A., Bahl D., Morris K., Stevens L.L., Haware R.V. (2020). Structure-mechanics and improved tableting performance of the drug-drug cocrystal metformin:salicylic acid. Eur.J. Pharm. Biopharm..

[9] Tomar S., Chakraborti S., Jindal A., Grewal M.K., Chadha R. (2020). Cocrystals of diacerein: Towards the development of improved biopharmaceutical parameters. Int. J. Pharm..

[10] Lee C., Cho A.Y., Yoon W., Yun H., Kang J.W., Lee J. (2019). Cocrystal Formation via Resorcinol–Urea Interactions: Naringenin and Carbamazepine. Cryst. Growth Des..

[11] Budziak-Wieczorek I., Macioek U. (2021). Synthesis and Characterization of a (-)-Epicatechin and Barbituric Acid Cocrystal: Single-Crystal X-ray Diffraction and Vibrational Spectroscopic Studies. ACS Omega.

[12] Abbas N., Latif S., Fatima K., Hussain A., Shamim R. (2021). Amelioration of physicochemical, pharmaceutical, and pharmacokinetic properties of lornoxicam by cocrystallization with a novel coformer. Drug Dev. Ind. Pharm..

[13] Bose P., Chandra S., Das A., Roy T., Mukherjee L. (2021). Enhancement of solubility of an oral hypoglycaemic drug, glimeperide by the technique cocrystallisation. J. Pharm. Sci..

[14] Vasilev N.A., Surov A.O., Voronin A.P., Drozd K.V., Perlovich G.L. (2021). Novel cocrystals of itraconazole: Insights from phase diagrams, formation thermodynamics and solubility. Int. J. Pharm..

[15] Yang D., Cao J., Jiao L., Yang S., Zhang L., Lu Y., Du G. (2020). Solubility and Stability Advantages of a New Cocrystal of Berberine Chloride with Fumaric Acid. ACS Omega.

[16] Is A., Svd B. (2019). Cocrystallization of carbamazepine with amides: Cocrystal and eutectic phases with improved dissolution. J. Mol. Struct..

[17] Kavanagh O.N., Wang C., Walker G.M., Sun C.C. (2021). Modulation of the powder properties of lamotrigine by crystal forms. Int. J. Pharm..

[18] Rai S.K., Gunnam A., Mannava M., Nangia A.K. (2020). Improving the Dissolution Rate of Anticancer Drug Dabrafenib. Cryst. Growth Des..

[19] Bommaka M.K., Mannava M.K.C., Suresh K., Gunnam A., Nangia A. (2018). Entacapone: Improving Aqueous Solubility, Diffusion Permeability, and Cocrystal Stability with Theophylline. Cryst. Growth Des..

[20] Almansa C., Mercè R., Tesson N., Farran J., Tomàs J., Plata-Salamán C. (2017). Co-crystal of Tramadol Hydrochloride–Celecoxib (ctc): A Novel API–API Co-crystal for the Treatment of Pain. Cryst. Growth Des..

[21] Gascon N., Almansa C., Merlos M., Vela J.M., Encina G., Morte A., Smith K., Plata-Salamán C. (2019). Co-crystal of tramadol-celecoxib: Preclinical and clinical evaluation of a novel analgesic. Expert Opin. Investig. Drugs.

[22] Guerain M., Guinet Y., Correia N.T., Paccou L., Hédoux A. (2020). Polymorphism and stability of ibuprofen/nicotinamide cocrystal: The effect of the crystalline synthesis method. Int. J. Pharm..

[23] Hiendrawan S., Veriansyah B., Widjojokusumo E., Soewandhi S.N., Wikarsa S., Tjandrawinata R.R. (2016). Physicochemical and mechanical properties of paracetamol cocrystal with 5-nitroisophthalic acid. Int. J. Pharm..

[24] Yin H.-M., Wu N., Zhou B.-J., Hong M.-H., Zhu B., Qi M.-H., Ren G.-B. (2021). Slow-Release Drug–Drug Cocrystals of Oxaliplatin with Flavonoids: Delaying Hydrolysis and Reducing Toxicity. Cryst. Growth Des..

[25] Wang J., Dai X.-L., Lu T.-B., Chen J.-M. (2021). Temozolomide–Hesperetin Drug–Drug Cocrystal with Optimized Performance in Stability, Dissolution, and Tabletability. Cryst. Growth Des..

[26] Wang L.-Y., Zhao M.-Y., Bu F.-Z., Niu Y.-Y., Yu Y.-M., Li Y.-T., Yan C.-W., Wu Z.-Y. (2021). Cocrystallization of Amantadine Hydrochloride with Resveratrol: The First Drug–Nutraceutical Cocrystal Displaying Synergistic Antiviral Activity. Cryst. Growth Des..

[27] Thipparaboina R., Kumar D., Chavan R.B., Shastri N.R. (2016). Multidrug co-crystals: Towards the development of effective therapeutic hybrids. Drug Discov. Today.

[28] Zhou J., Li L., Zhang H., Xu J., Huang D., Gong N., Han W., Yang X., Zhou Z. (2020). Crystal structures, dissolution and pharmacokinetic study on a novel phosphodiesterase-4 inhibitor chlorbipram cocrystals. Int. J. Pharm..

[29] Dhillon S. (2015). Palbociclib: First global approval. Drugs.

[30] Gopalan P.K., Pinder M.C., Chiappori A., Ivey A.M., Villegas A.G., Kaye F.J. (2014). A phase II clinical trial of the CDK 4/6 inhibitor palbociclib (PD 0332991) in previously treated, advanced non-small cell lung cancer (NSCLC) patients with inactivated CDKN2A. J. Clin. Oncol..

[31] Jin H.P., Park H., Kim K.H., Kim J.S., Choi I.S., Roh E.Y., Ji E.K., Chang M. (2020). Acute Lymphoblastic Leukemia in a Patient Treated with Letrozole and Palbociclib. J. Breast Cancer.

[32] Xu Y., Yao Y., Park W.D., Derebail S., Munshi N.C. (2020). Enhancing the Immune Surveillance in Multiple Myeloma Via CDK4/6 Inhibition. Blood.

[33] Tamura K. (2019). Differences of cyclin-dependent kinase 4/6 inhibitor, palbociclib and abemaciclib, in breast cancer. Jpn. J. Clin. Oncol..

[34] Kwapisz D. (2017). Cyclin-dependent kinase 4/6 inhibitors in breast cancer: Palbociclib, ribociclib, and abemaciclib. Breast Cancer Res. Treat..

[35] Sun W., Klamerus K.J., Yuhas L.M., Pawlak S., Plotka A., O’Gorman M. (2017). Impact of acid-reducing agents on the pharmacokinetics of palbociclib, a weak base with ph-dependent solubility, with different food intake conditions. Clin. Pharm. Drug Dev..

[36] Xu H., Yu S., Liu Q., Yuan X., Mani S., Pestell R.G., Wu K. (2017). Recent advances of highly selective CDK4/6 inhibitors in breast cancer. J. Hematol. Oncol..

[37] Rocca A., Farolfi A., Bravaccini S., Schirone A., Amadori D. (2014). Palbociclib (PD 0332991): Targeting the cell cycle machinery in breast cancer. Expert Opin. Pharm..

[38] Xu W., Shi X.-S., Tian F., Anne Z., Chen Q., Huang L.-N., Hong G. (2018). Palbociclib Amorphous. State. Patent.

[39] Aimei S. (2018). Novel Palbociclib Crystal. Form. Patent.

[40] Lui Z.-T., Li X.-F., Geng Q., Xue Y. (2020). Palbociclib Pharmaceutical Composition and Method for Preparing. Same. Patent.

[41] Zhang M., Xiong X., Suo Z., Hou Q., Gan N., Tang P. (2019). Co-amorphous palbociclib–organic acid systems with increased dissolution rate, enhanced physical stability and equivalent biosafety. RSC Adv..

[42] Cho M.-Y., Kim P., Kim G.-Y., Lee J.-Y., Song K.-H., Lee M.-J., Yoon W., Yun H., Choi G.J. (2017). Preparation and Characterization of Aripiprazole Cocrystals with Coformers of Multihydroxybenzene Compounds. Cryst. Growth Des..

[43] Paul M., Desiraju G.R. (2019). From a Binary to a Quaternary Cocrystal: An Unusual Supramolecular Synthon. Angew. Chem. Int. Ed..

[44] Shimpi M.R., Alhayali A., Cavanagh K.L., Rodríguez-Hornedo N., Velaga S.P. (2018). Tadalafil–Malonic Acid Cocrystal: Physicochemical Characterization, pH-Solubility, and Supersaturation Studies. Cryst. Growth Des..

[45] Liu W., Ma R., Liang F., Duan C., Zhang G., Chen Y., Hao C. (2021). New Cocrystals of Antipsychotic Drug Aripiprazole: Decreasing the Dissolution through Cocrystallization. Molecules.

[46] Bofill L., Sande D.D., Barbas R., Prohens R. (2021). A New and Highly Stable Cocrystal of Vitamin D3 for Use in Enhanced Food Supplements. Cryst. Growth Des..

[47] Sheldrick G.M. (2008). A Short History of ShelX. Acta Crystallogr. A.

[48] Neese F., Wennmohs F., Becker U., Riplinger C. (2020). The orca quantum chemistry program package. J. Chem. Phys..

[49] Spackman M.A., Jayatilaka D. (2009). Hirshfeld surface analysis. CrystEngComm.

[50] Abidi S., Azim Y., Gupta A.K., Pradeep C.P. (2017). Mechanochemical synthesis and structural characterization of three novel cocrystals of dimethylglyoxime with n-heterocyclic aromatic compounds and acetamide. J. Mol. Struct..

[51] Hong M., Li S., Ji W., Qi M.H., Ren G.B. (2021). Cocrystals of lenvatinib with sulfamerazine and salicylic acid: Crystal structure, equilibrium solubility, stability study, and anti-hepatoma activity. Cryst. Growth Des..

[52] Cremer D., Pople J.A. (1975). General definition of ring puckering coordinates. J. Am. Chem. Soc..

[53] Zhang Y.X., Wang L.Y., Dai J.K., Liu F., Li Y.T., Wu Z.Y. (2019). The comparative study of cocrystal/salt in simultaneously improving solubility and permeability of acetazolamide. J. Mol. Struct..

[54] Dhumal R.S., Kelly A.L., York P., Coates P.D., Paradkar A. (2010). Cocrystalization and simultaneous agglomeration using hot melt extrusion. Pharm. Res..

[55] Amo E.M., Vellonen K.S., Kidron H., Urtti A. (2015). Intravitreal clearance and volume of distribution of compounds in rabbits: In silico prediction and pharmacokinetic simulations for drug development. Eur. J. Pharm. Biopharm..

