# Supplementary material for: Two Novel Palbociclib-Resorcinol and Palbociclib-Orcinol Cocrystals with Enhanced Solubility and Dissolution Rate
Source: Pharmaceutics. 2021 Dec 23;14(1):23. doi: 10.3390/pharmaceutics14010023 (PMC8781472; doi:10.3390/pharmaceutics14010023)
Supplement: Supplementary file 1 [file pharmaceutics-14-00023-s001.zip › pharmaceutics-1477120-supplementary.pdf]

Supplementary Information: Two Novel Palbociclib-Resorcinol and Palbociclib-Orcinol Cocrystals with Enhanced Solubility and Dissolution Rate

Chenxin Duan, Wenwen Liu, Yunwen Tao, Feifei Liang, Yanming Chen, Xinyi Xiao, Guisen Zhang, Yin Chen and Chao Hao

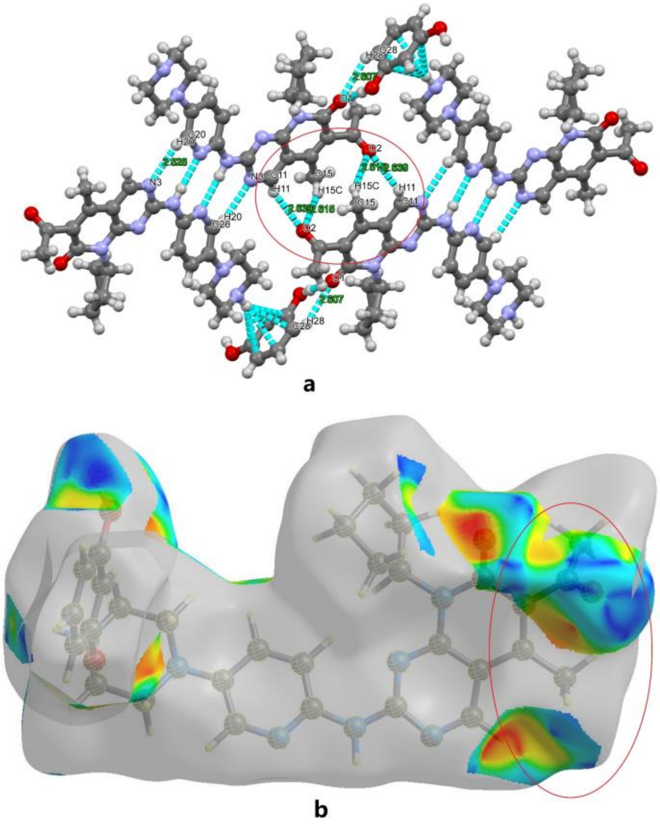

Figure S1. (**a**) C-H· · ·O interaction between PAL-RES molecules made by Mercury 4.3.0 software, (**b**) shape-index surface for C-H· · ·O interaction of PAL-RES through Hirshfeld Surface Analysis.

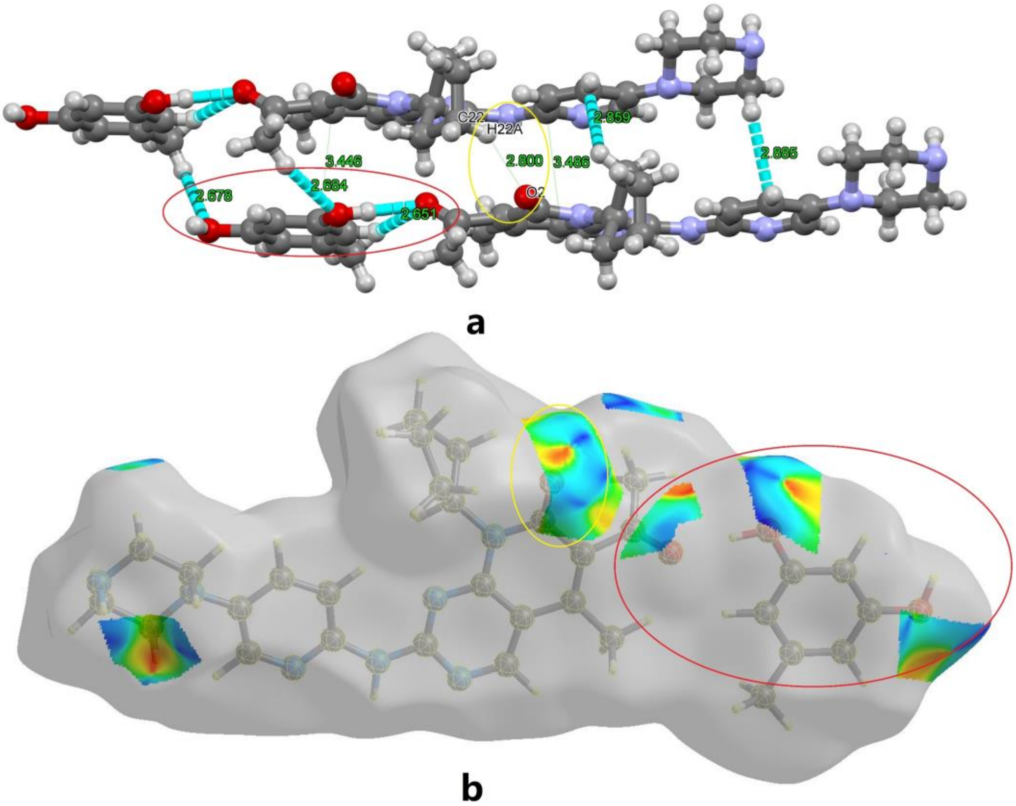

Figure S2. (**a**) C-H· · ·O and O-H· · ·O interactions between PAL-ORC molecules made by Mercury 4.3.0 software, (**b**) shape-index surface for C-H· · ·O and O-H· · ·O interactions of PAL-ORC through Hirshfeld Surface Analysis. (Yellow circles: C-H· · ·O; Red circles: O-H· · ·O).

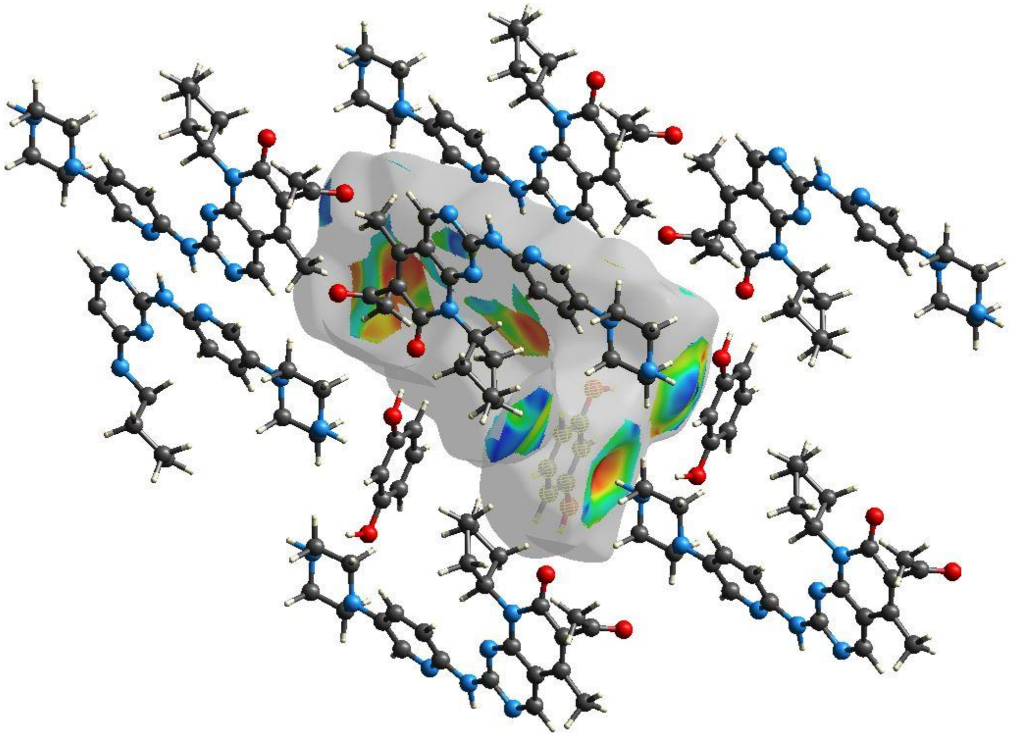

Figure S3. C-H· · ·π interaction between PAL-RES molecules.

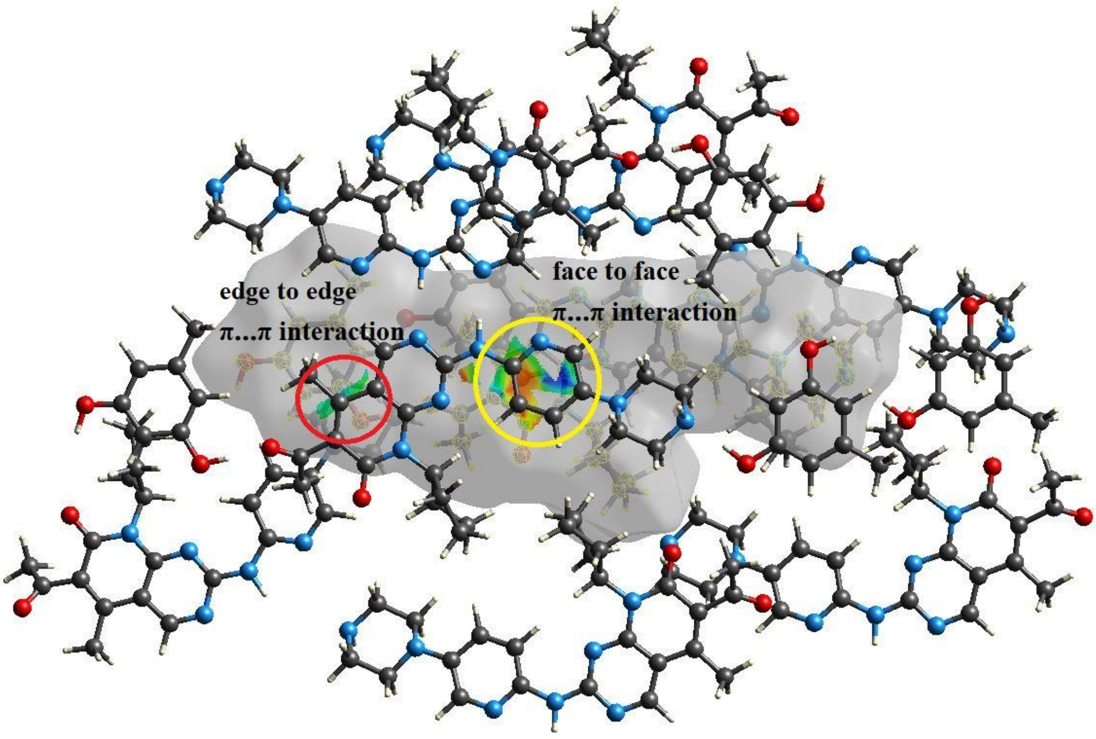

Figure S4. π· · ·π interaction between PAL-ORC molecules.

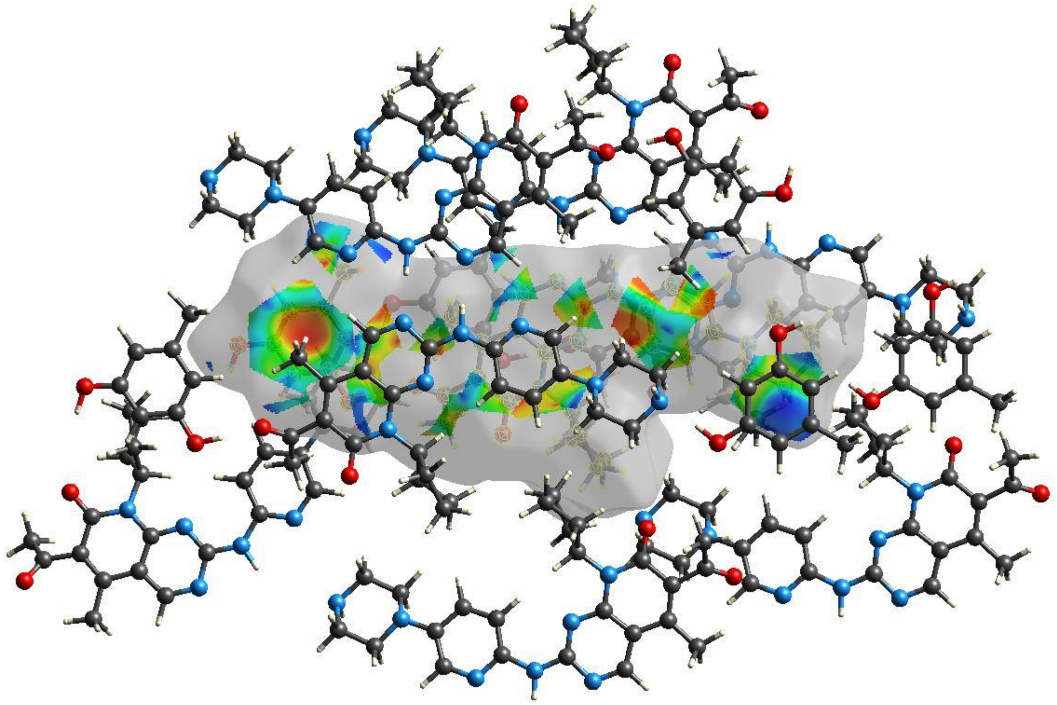

Figure S5. C-H· · ·π interaction between PAL-ORC molecules.

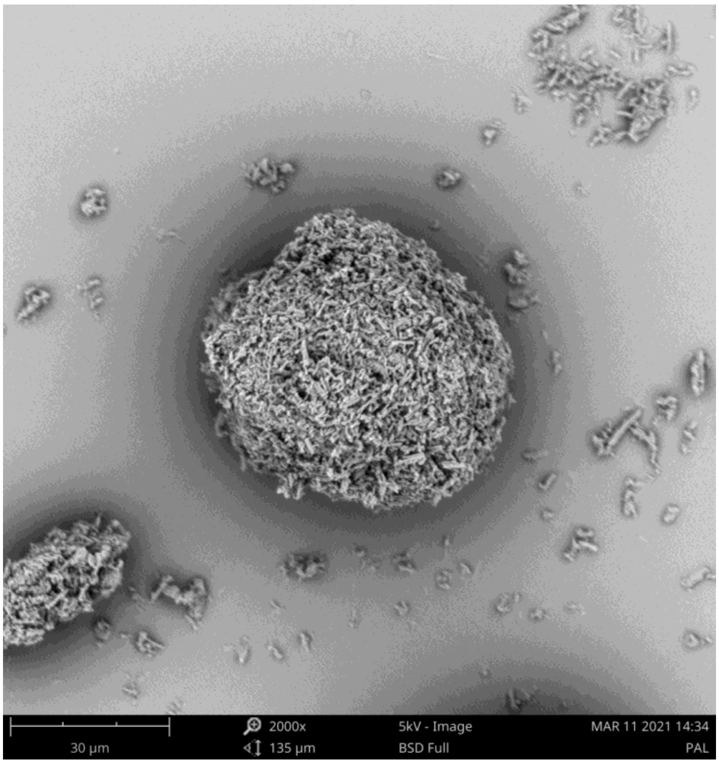

Figure S6. SEM images of PAL in 2000×.

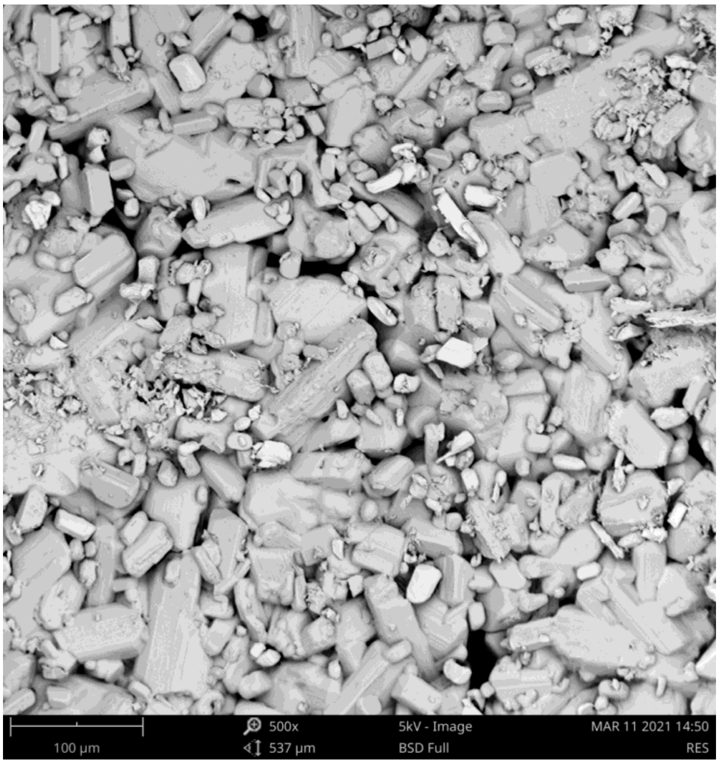

Figure S7. SEM images of RES in 500×.

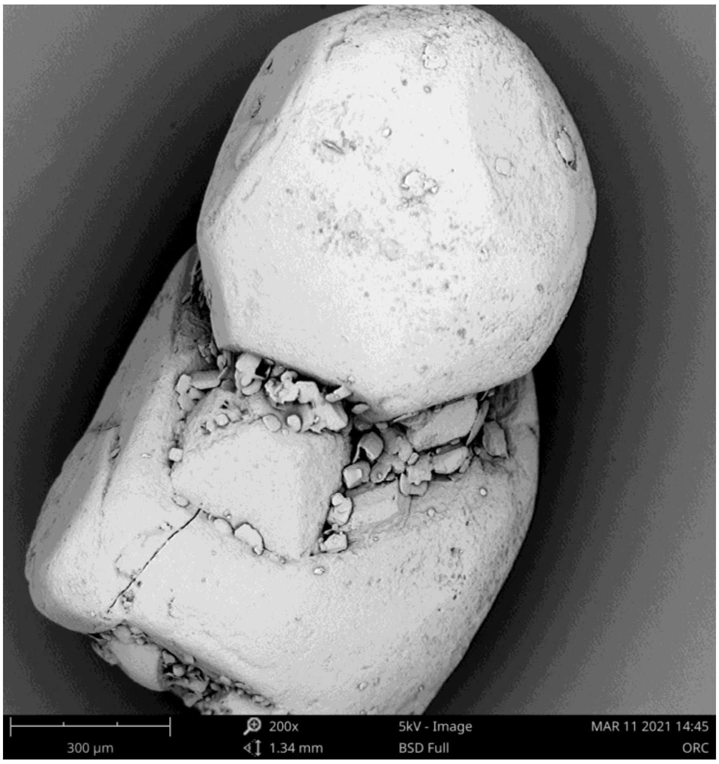

Figure S8. SEM images of ORC in 200×.
